# Treatment of Fractures in a Country Practice*Read before the East Texas Medico Chirurgical Society, November meeting, 1902.

**Published:** 1903-06

**Authors:** W. B. Collins

**Affiliations:** Lovelady, Texas


For Texas Medical Journal.

### Treatment of Fractures in a Country Practice.*

BY W. B. COLLINS, M. D., LOVELADY, TEXAS.

*Mr. President and Fellow Practitioners:*

You will pardon me for coming before you with a paper, which, from its title, would imply that doctors in the country should treat their patients differently from doctors in the towns. Such, however, is not my intention. We who practice in the country well know the great difficulties which we encounter in our efforts to do scientific surgical work. We miss the trained nurse, aseptic rooms, consulting surgeons, improved appliances and dressing so necessary to complete success, etc. In fact, the wonder is that we are as successful as we are. Having practiced in the country for a number of years, I thought possibly I might throw from the rag-bag of my experience something which would interest some of you. My remarks are in a general way, and I will only make some suggestions from my individual experience and report a case, which will show the necessity of giving this subject, viz.: treatment of fractures, a great deal more attention that it often receives. Any violence applied to the bones results in lesions analogous to those produced by the same cause on the soft tissues. Fractures are the counterparts of wounds of the soft parts, and demand immediate care either in operative procedures or in the employment of adjusting and retaining appliances. Fractures are quite common, and, according to Bruns, they make up more than a seventh part of all injuries which come under our observation, and are about ten times more common than dislocations.

I will divide them, for practical purposes (as does Smith), into the simple, the compound, and the shot fractures.

In the simple fractures the bone is broken at a single point; the lesion is subcutaneous, and no other important parts are involved; it is, therefore, in the most favorable condition for repair.

A fracture is compound when it communicates through a wound with the external air. These injuries have always been regarded

*Read before the East Texas Medico Chirurgical Society, November meeting, 1902.

as dangerous, because such wounds commonly inflame and suppurate. Projectiles cause a variety of partial and complete fractures. The former are: First, removal of a portion of bone; second, splintering off of fragments of the external cylindrical part of a bone; third, making a hole throughout the entire substance of the bone; fourth, driving the external cylinder into the cancellated structures. The latter, first, simple when the injury is indirect; and second, compound when the projectile is brought in direct contact with the injured bone. These fractures are always serious injuries, as they frequently involve the question of resection and amputation, and are always liable to dangerous complications, as hemorrhage, tetanus, septicemia, pyemia. The course of treatment indicated varies with the bone fractures and the nature and extent of the injury. Upon being called to treat a patient suffering with fracture, we should endeavor to inspire him with confidence that he is not to be unnecessarily hurt. Sit quietly beside him, and inquire minutely into all the circumstances relating to the accident. Note carefully the condition of his heart action and temperature. If suffering materially from shock, administer one-fourth grain morphia, 1-150 grain sulph. atropia (hypodermically); also warm apartments, and apply artificial heat to extremities. When shock is very severe, strychnia, 1-30 grain, hypodermically, and brandy or whisky by mouth. Be prompt to arrest hemorrhage where severe. Remove all clothing from the injured limb, with the utmost care. Notice its position, contour, points of abrasion, discoloration, or swelling. Pass the fingers lightly along the surface of the limb; pressing more firmly at points where there are appearances of injury. Finally, to solve all doubts, grasp the limb so as to make traction of the lower fragment, rotate to obtain crepitus, and make lateral motions to indicate the false point of motion. In the application of the necessary dressings, let gentleness and a manifest regard for the patient's suffering characterize every act, and throughout the subsequent treatment of the case proceed slowly, thoughtfully and systematically, for rude and awkward manipulation, by which pain is needlessly inflicted, are frequent sources of inflammation, suppuration and gangrene.

The signs of a fracture, on which reliance can be placed, are: First, crepitus, obtained by rotating the lower fragment; second, preternatural mobility, produced by lateral movements of the fragments; third, spontaneous displacement when reduction of the fragments has been effected. The treatment is, of course, replacement of the fragments and maintenance of their extremities in apposition. Replacement should be effected as soon as possible after the accident. The fragments may usually be placed in coaptation by extension and counter-extension with the hands; but, should such means fail, anesthetics should be used, and even pulleys.

Maintenance is accomplished by side or coapting splints, by long or extending splints, by weight and pulley, by plastic apparatus, or by combination of these methods.

My selection of a dressing would depend entirely upon the character and location of the fracture. In simple fracture of the long bones, with slight contusion or abrasions, a plaster paris bandage, carefully applied, would be my preference; but, should there exist any marked degree of contusion or swelling, from any cause, avoid the plaster until you are satisfied that healthy repair is well advanced. Employ any of the ordinary methods of fixation, but preferably those which are easily removed and will allow frequent examination. In this connection I will tell you of a case in which no union took place. An extensive suppuration, which came near costing the patient his limb, resulted from too early employing the plaster dressing.

About two years ago I was called to a saw-mill town by a physician, who told me, by 'phone, that he desired me to do an amputation upon one of his patients. The patient was a negro man, about 25 years old; no constitutional taint, and of robust build. My friend, Dr. F. L. Barnes, accompanied me to see the man. The doctor gave us this history of the case: About twenty-four days prior to our visit, the negro had jumped from a moving hand-car and sustained a simple fracture of both bones of the leg, about the juncture of the middle and lower third. There was considerable abrasion of the skin, though the parts were not lacerated, so far as could be seen. The doctor at once reduced the fracture and secured it by plaster paris bandage. This was applied very close, and completely prevented any ocular or manual examination of the limb. If I am not mistaken, this dressing was allowed to remain until a few days before our visit; the doctor and patient both confident that repair was going on satisfactorily. Imagine the doctor's feelings when, upon removing the bandage, he found extensive suppuration. The early abrasions were now large, ugly ulcers, and, of course, no bony union. The fracture was now in worse condition than at first. The patient had run the gauntlet of septicemia, pyemia, secondary hemorrhage, gangrene, etc. The doctor decided upon amputation, and hence our visit. After carefully examining the injury, we decided to make further effort to save the limb, which I am gratified to tell you, was a decided success. We begun by cleansing the whole limb, and especially from the knee down, very thoroughly by scrubbing with soap and water first, then a solution bi-chloride mercury, 1 to 1,000; freely incised the sinus in front, and made a counter-opening on back of leg, which was followed by liberal discharge of pus. We then passed a dissector between the bones and effected a direct channel for drainage from front to back of leg. We next irrigated every niche and pocket in the wound; then nipped off the ebonized ends of the bones, as best we could with forceps and curette, and after again irrigating with sterile warm water, proceeded to dress by putting a good, gauze drain through the leg, dusting freely with iodoform and covering entire diseased portion of limb with plain sterile gauze; coapted ends of the bones and confined with cotton and heavy bandage, reinforced at site of fracture with pasteboard splints; placed limb in an ordinary fracture box and put him on a rich and nutritious diet. This negro made an uneventful and, considering all things, a rapid recovery. Now, the safer plan would have been to have placed this limb in a well-padded fracture box, examined it from time to time, and upon the least suspicion of suppuration in the bones or soft parts freely incised under antiseptic precautions and freely drained the wound. This would have been a time-saving and in some instances a life-saving procedure.

Now, gentlemen, I will not bore you further. I have in a very general way outlined the treatment of all fractures when I say carefully note the kind of injury to be treated, employ such means as will best secure rest, fixation, asepsis, promote good circulation, and preserve the symmetry and contour of injured parts, and finally remember that conservatism and eternal vigilance are the price of success. Rely on yourselves as far as possible in all after-dressings. Visit your patient when you can, and satisfy yourself at each visit that all is going well.

